# Tradition, taste and taboo: the gastroecology of maternal perinatal diet

**DOI:** 10.1136/bmjnph-2021-000252

**Published:** 2021-07-05

**Authors:** Hannah G Lunkenheimer, Oskar Burger, Santosh Akhauri, Indrajit Chaudhuri, Lisa Dibbell, Faiz A Hashmi, Tracy Johnson, Emily E Little, Sudipta Mondal, Nachiket Mor, Neela Saldanha, Janine Schooley, Cristine H Legare

**Affiliations:** 1 Department of Psychology, The University of Texas at Austin, Austin, Texas, USA; 2 Project Concern International, San Diego, California, USA; 3 Bill & Melinda Gates Foundation, Seattle, Washington, USA; 4 The Banyan Academy of Leadership in Mental Health, Thiruvidanthai, Tamil Nadu, India; 5 Centre for Social and Behaviour Change, Ashoka University, Sonepath, Haryana, India

**Keywords:** malnutrition, nutrient deficiencies, dietary patterns

## Abstract

**Background:**

Maternal malnutrition is a major source of regional health inequity and contributes to maternal and infant morbidity and mortality. Bihar, a state in eastern India adjacent to Jharkhand and West Bengal, has relatively high neonatal mortality rates because a large portion of infants are born to young mothers. Bihar has the second-highest proportion of underweight children under 3 in India, with infant mortality rates of 48 per 1000 live births. Maternal malnutrition remains a major threat to perinatal health in Bihar, where 58.3% of pregnant women are anaemic.

**Methods:**

We examined dietary beliefs and practices among mothers, mothers-in-law and community members, including Accredited Social Health Activists (ASHAs), using focus group discussions (n=40 groups, 213 participants), key informant interviews (n=50 participants) and quantitative surveys (n=1200 recent mothers and 400 community health workers). We report foods that are added/avoided during the perinatal period, along with stated reasons underlying food choice. We summarise the content of the diet based on responses to the quantitative survey and identify influencers of food choice and stated explanations for adding and avoiding foods.

**Key findings:**

Analyses for all methodologies included gathering frequency counts and running descriptive statistics by food item, recommendation to eat or avoid, pregnancy or post partum, food group and health promoting or risk avoiding. During pregnancy, commonly added foods were generally nutritious (milk, pulses) with explanations for consuming these foods related to promoting health. Commonly avoided foods during pregnancy were also nutritious (wood apples, eggplant) with explanations for avoiding these foods related to miscarriage, newborn appearance and issues with digestion. Post partum, commonly added foods included sweets because they ease digestion whereas commonly avoided foods included eggplants and oily or spicy foods. Family, friends, relatives or neighbours influenced food choice for both mothers and ASHAs more than ASHAs and other health workers.

Perinatal dietary beliefs and behaviours are shaped by local *gastroecologies* or systems of knowledge and practice that surround and inform dietary choices, as well as how those choices are explained and influenced. Our data provide novel insight into how health influencers operating within traditional and biomedical health systems shape the perinatal dietary beliefs of both mothers and community health workers.

**What this paper adds**

- Perinatal gastroecologies are the systems of knowledge and practice that surround and inform dietary choices during pregnancy and postpartum, including how those choices are explained and influenced.
- Across the perinatal period, most added and avoided foods were nutritious. Common explanations included promoting health and avoiding risk.
- Accredited Social Health Activists (ASHAs) and the beneficiaries they serve engage in similar dietary practices, which should inform the dietary training ASHAs receive.

## Introduction

The perinatal period is associated with unique nutritional needs that have direct implications for maternal and child health. Perinatal dietary beliefs and behaviours are shaped by local *gastroecologies*. Gastroecologies are the systems of knowledge and practice that surround and inform dietary choices, including how those choices are explained and influenced. They are increasingly the product of both traditional medicine and modern biomedicine, which can complement or conflict with each other. According to the World Health Organization, traditional medicine refers to the ‘knowledge, skill, and practices based on the theories, beliefs, and experiences indigenous to different cultures, used in the maintenance of health and in the prevention, diagnosis, improvement, or treatment of physical and mental illnesses’.^1^ In contrast, modern biomedicine provides a framework for understanding the interaction of genes and environment, how these produce individual characteristics and social group formation and how these interactions lead to pathophysiology and diseases.^2^ Whereas traditional medicine and biomedicine as part of a complex health system provide health recommendations, a distinguishing characteristic is that biomedicine relies on the scientific method to identify aetiology and evaluate treatment efficacy. Introducing biomedical beliefs and practices does not replace traditional medicine^3–6^; instead, they coexist and jointly influence decision-making about maternal perinatal diet.^7^ Thus, a comprehensive understanding of the perinatal diet requires documenting complex gastroecologies of dietary beliefs and practices.

Maternal perinatal gastroecologies vary within and between populations, resulting in different beliefs about the same food or foods of the same nutritional value. They also vary based on the perinatal period.^8^ Variation in nutritional practices is due to food availability, local beliefs and recommendations from respective health influencers. While food taboos in perinatal diet have been documented in previous literature, many researchers capture data from a single perspective. For example, similar studies in Bihar and other Indian states detail foods mothers avoid eating during pregnancy and provide explanations for avoiding these foods.^9 10^ To understand complex gastroecologies, however, we argue that the entire network of traditional and biomedical health influencers must be examined. A key feature of our approach is to use mixed methods to examine the role of a diverse set of health influencers operating within (and between) traditional and biomedical belief systems in shaping beliefs about perinatal diet.

Local gastroecologies impact the dietary choices of women worldwide during one of the most critical periods of the human life course, yet their influence on health and nutrition remains poorly understood from a global health perspective. The current research documents and describes the gastroecology of maternal perinatal diet among mothers and Accredited Social Health Activists (ASHAs) in Bihar, a state in eastern India with persistent perinatal malnutrition.^11^ Bihar is India’s most rural and economically poorest state, with over 88% living in rural areas and a gross state domestic product per person that is the lowest of all 36 states and union territories.^12^ While effort has been made by Integrated Child Development Services and the National Rural Health Mission of India^13 14^ to improve health and nutrition service delivery, about 40% of pregnant women of Bihar did not receive any form of antenatal care in 2015–2016. There is substantial variation in antenatal healthcare delivery in Bihar.^15^ For example, 33.2% of mothers received supplemental food while pregnant, whereas only 18.4% received nutrition education. Similar rates of service delivery were reported for mothers post partum. Increasing the consumption of iron and folic acid among pregnant women in Bihar is critical as rates of anaemia exceed 57% and breastfeeding women are even more at risk.

Bihar has relatively high neonatal mortality rates because a large portion of infants are born underweight, who are more likely to be born to younger, underweight mothers.^16^ Bihar has the second-highest proportion of underweight children under 3 in India, with infant mortality rates of 48 per 1000 live births.^17^ Maternal malnutrition remains a major threat to perinatal health in Bihar where 58.3% of pregnant women are anaemic.^17^ Sufficient intake of micronutrients such as iron and folic acid is critical for the development of the fetus in utero and the mother and infant health post partum.

Previous researchers used Food and Agriculture Organization (FAO) of the United Nations guidelines to study dietary diversity and variation in dietary diversity scores (DDS) from adolescent girls, pregnant women and mothers in Bihar, Chhattisgarh and Odisha.^10^ High daily consumption of grains and tubers was reported for almost all participants. Vegetables, yellow fruits/vegetables and pulses and beans were consumed by most respondents. Low levels of consumption were reported for nuts and seeds, eggs and dairy milk/milk products. The mean DDS for pregnant in Bihar was 4.05, and over half of the sampled population did not meet the mean dietary diversity goal of consuming at least five food groups daily. Furthermore, the dietary diversity of all target groups in Bihar was poorer than in the other two studied states.^10^

Many factors contribute to these trends in daily nutrition for Indian states such as Bihar. High production and low pricing of rice and processed cereals may provide easier accessibility for families to afford. Moreover, some researchers claim that dietary recommendations are not affordable for most households.^18^ Availability of foods such as legumes and vegetables is not produced at high enough rates to meet the demand as a main source of plant-based protein in India.^19^ These factors prove great difficulty for women to achieve optimal nutritional intake even before conception.

### Biomedical recommendations for perinatal diet

Biomedical dietary recommendations typically concern caloric amounts or nutritional content. Mothers should increase caloric intake by approximately 300 calories during pregnancy to keep up with the metabolic demands of gestation and 450–500 kcal during lactation.^20 21^ Guidelines include a variety of fruits, vegetables, dairy, meat/poultry/fish, grains and tubers, eggs, pulses and nuts/seeds^22^ that contain critical macronutrients and micronutrients from food and increasingly from supplements.^23^ Deficits in these essential micronutrients can result in severe health risks to both mother and infant including maternal anaemia and preterm birth.^24^ Eating a well-balanced diet promotes general health and reduces pregnancy-related symptoms such as nausea and fatigue.^25^ Post partum, foods associated with increased milk production such as papaya, ginger, leafy greens and whole grains are recommended. Recommended foods to avoid during pregnancy and post partum include certain types of seafood, raw milk products, raw protein, alcohol, caffeine and foods with higher risks of microbial contamination.^21^

### Traditional medical recommendations for perinatal diet

Traditional medicine is a system of health-related recommendations and practices based on the beliefs and experiences indigenous to a cultural group.^1^ Traditional medicine influences dietary choices around the world and, like biomedicine, operates to improve health and avoid risk.^7 26^ In India, Ayurveda and other traditional systems of medicine are widely practised and predate biomedicine.^27^ In rural dominant states like Bihar, approximately 70% of the population relies on Ayurveda.^28^ We discuss Ayurveda as a prominent form of traditional medicine in this region.

Ayurvedic dietary principles are based on the concept of heating/cooling properties, ease of digestion and *doshas* (energies that balance the body and mind). To achieve dietary balance, Ayurveda extends the humoral concept of food temperature: acidic and salty foods are considered ‘hot’, and sweet foods, vegetables and fruits are considered ‘cold’.^29^ Pregnancy is considered a ‘hot’ state, and thus temporal balance may be achieved by consuming ‘cold’ foods.^30–33^ Hot foods, such as papaya, are generally avoided during pregnancy because they are associated with increased risks of miscarriage and abortion. Similarly, ‘cold’ foods are avoided during lactation because of concerns they may affect the quality and quantity of milk production.^34 35^ New mothers are encouraged to consume milk, ghee (clarified butter), nuts and jaggery (concentrated cane sugar) to return to a state of balance.

Ayurveda also describes digestive and metabolic power controlled by the individual known as *agni*.^36^ The three *doshas* or energies (vata: movement, pitta: digestion and metabolism, kapha: lubricates joints) that define a person must be balanced through six tastes, known as *rasas*. These tastes (salty, sweet, bitter, astringent, pungent and sour) all play a role in stimulating the agni. Whereas sweets are believed to promote overall strength, sour and pungent foods stimulate proper digestion. During pregnancy, however, the pitta dosha is aggravated by sour and pungent foods. Salty and oily foods can cause water retention whereas astringent foods can cause constipation, so avoiding these rasas is recommended. Warm foods are also believed to stimulate digestion. General Ayurvedic recommendations for perinatal diet include avoiding heating foods (sour, pungent, salty) during pregnancy, adding cooling foods (bitter, sweet, astringent) during pregnancy and adding easy-to-digest foods post partum to reinvigorate agni after delivery. In sum, Ayurveda’s guidelines focus on content and kind of food, tend not to specify the amount of food to consume and make personalised recommendations based on the individual’s state and overall diet.^37^

### Health influencers

Gastroecologies are shaped by a variety of influencers that interact in a complex healthcare system. These include traditional health influencers such as birth attendants (dais), religious authorities in the community (eg, Pandits, Maulvis), family, relatives, neighbours and biomedical health influencers such as community health workers (CHWs). Table 1 provides definitions of the health influencer terminology in our study.

**Table 1:** Health influencer key

CHW	Community Health Workers	Local health educators who deliver health information and services to communities.
ASHA	Accredited Social Health Activist	CHWs who help connect mothers to the formal healthcare system, including nutritional information.
AWW	Anganwadi Worker	CHWs who provide nutritional and health education services to families.
AWC	Anganwadi Centre	A rural childcare centre. AWCs define the catchment areas for ASHAs and AWWs. Anganwadi means ‘courtyard shelter’.
ANM	Auxiliary Nurse Midwife	CHWs based in health subcentres in India who first connect the community with health services. ANMs provide training and support for AWWs and ASHAs. The areas serviced by health subcentres are larger than AWCs and there may be 4–5 ASHAs (and AWCs) per ANM.
GovDoc	Government doctor	Professionally trained medical doctors working in the government healthcare system of India.
PrivClin	Private clinic	Privately owned facilities that exist in parallel to the government health facilities.
SHG	Self-help groups	Local community groups that provide educational and social support for members, typically meeting on a monthly basis.
FrRelNeigh		Friends, family members, neighbours, other relatives
RMP*	Rural Medical Practitioner	Uncertified healthcare providers who nominally practise biomedicine but lack a formal approval or certification.
Pandit*		A Hindu priest.
Maulvi*		A Muslim religious leader.
Dai*		Traditional midwives who help mothers through pregnancy and provide support and preparation for delivery.

*Dais are traditional birth attendants with a role similar to a midwife. Dais are not certified or trained by the government; however, they have extensive experience with the birthing process and learn through intergenerational knowledge. RMPs are another potential source of health-relevant information in Bihar. RMPs lack formal healthcare accreditation but often have some biomedical training. Religious leaders may also be consulted by women on a range of topics including rituals associated with the perinatal period. Pandits and Maulvis are, respectively, Hindu and Muslim religious leaders. There is a sizeable Muslim population in Bihar; however, 83% of the population practises Hinduism.^37^

CHWs including ASHAs, Anganwadi workers (AWWs) and auxiliary nurse midwives (ANMs) serve the vital role of connecting local communities to the government healthcare system. CHWs are part of the health system and support perinatal health initiatives, including providing product and information-oriented health services^15^ such as food supplements and dietary guidance. ASHAs connect pregnant mothers from their community to the government-regulated health system with duties including pregnancy registration, administering vaccines, distributing iron-folic acid (IFA) tablets, regular home visits and accompanying mothers to a clinic or hospital to give birth.

The nature of the advice given and the likelihood that the advice is followed vary based on a complex suite of individual-level and group-level factors. Research on cultural transmission of health practices through social norms has shown that vertical transmission (primarily parent to offspring) is particularly important for dietary decisions about which foods to avoid.^38^ Placek *et al. (2017)* found in rural Southwest India that mothers, grandmothers and mothers-in-law (MIL) were responsible for influencing 76% of reported dietary avoidances.^9^ They also found that other origins of food taboos included individual aversions that spread by peer-to-peer cultural transmission in group settings.

### Objectives

Our objective is to document the maternal perinatal gastroecology in Bihar, including both traditional and biomedical perspectives and explanations of health influencers, ASHAs and mothers. We use a mixed methods approach to describe dietary content and quantify individual-level influences on dietary choices and reasoning. First, we describe the content of the perinatal diet in terms of frequencies of foods that are avoided or added, emphasising associations with the stated reason behind the food choice and the source of information for why the choice was made. We also report variation in the number and types of foods avoided by mothers and ASHAs. Second, we summarise the content of the diet as revealed by responses to the quantitative questionnaire and identify which health influencers and stated reasons for food choice are most prominent for adding and avoiding foods. Finally, we highlight the benefits of using our mixed methods approach to study this complex gastroecology and list how local perinatal diet can be leveraged to improve maternal and infant health outcomes.

## Methods

### Qualitative study design and participants

The qualitative discussions consisted of focus group discussions (FGDs) and key informant interviews (KIIs) with rural women from Bihar and select health influencers. For the FGDs, participants were recruited from 21 Anganwadi centres (AWCs) within three blocks, randomly selected within the districts (4 villages in the blocks of Biharsharif in the Nalanda district, 6 from the Rajgir block of Nalanda district and 11 from the Warisnagar block of Samastipur district). Villages were selected based on geographic relationship to urban areas. Participants were purposefully chosen based on their age, caste, religion and dialect. Our overall study focuses on the role of the ASHA and the other respondents are included to obtain a wider understanding across the many sources of influence on perinatal health decisions.

Twenty FGDs were conducted with two groups of independently recruited women, recent mothers (n=107) and MILs (n=106), for a total of 40 FGDs. For the recent mother sample, women had to have given birth within the previous 2 years (as opposed to within 6 months for the quantitative survey). MILs were older mothers who have a daughter-in-law with a child under 2 years of age. The participants in the mother FGDs ranged in age from 18 to 35 with a mean age of 24, and in the MIL FGDs ranged from 35 to 75 with a mean age of 52. The mother FGDs were composed of 69 Hindu and 38 Muslim women. The MIL FGDs had 68 and 37, respectively. Most of the FGDs had exclusively Hindu or Muslim participants.

Fifty KIIs were conducted, 12 of which were with ASHAs, 11 with AWWs in addition to 10 dais, 6 rural medical practitioners, 5 Pandits and 6 Maulvis (see table 1). The participants in KIIs ranged in age from 24 to 65 years with a mean of 49. While the focus of the KIIs was on actual behaviours, the free-flowing and semistructured components of the discussion brought occasional variations in interpretation between recommendation and practice.

### Methods for qualitative studies

FGDs and KIIs were both set up as semistructured interviews that encouraged open conversation. Investigators provided a structure whereby they would ask about different kinds of health-related beliefs, rituals and practices (including food choices) during the perinatal period (from 2 months before conception to 6 months post partum). We documented food-related restrictions and recommendations, as well as the participant’s self-reported views and beliefs about their dietary practices, their explanations and expected outcomes. A systematic checklist was developed via piloting and then provided to trained local Project Concern International investigators who conducted the interviews. The checklist served as a guide to ensure that major topics were covered in each FGD while leaving ample room for free-flowing discussion.

Demographic information was recorded on all participants. Researchers used a standardised set of checklists and followed uniform guidelines to ensure a consistent dialogue across FGDs. Follow-up questions were used to resolve ambiguity and ensure the dialogue was free flowing. The researchers collected data for 3 weeks in January 2019. The FGDs consisted of four to seven participants, in addition to the trained researcher and a note taker, while KIIs consisted of a trained researcher, the interviewee and a note taker.

### Quantitative study design and participants

For our quantitative survey, the survey team divided Bihar into three zones based on the three main dialects of Hindi spoken in Bihar (Bhojpuri, Magahi and Maithili). One of these three linguistic areas is much larger than the other two (Maithili). Hence, in our sampling design we selected two districts from Maithili and one each from Magahi and Bhojpuri. Within each of these four districts, two blocks were randomly sampled, and from within each of the eight selected blocks, 50 AWCs were randomly sampled. AWCs were the focal sampling unit because these represent the catchment areas for ASHAs. Our participant recruitment included one ASHA and three recent mothers (who had given birth within the previous 6 months) from each of these 400 AWCs for a total of 400 ASHAs and 1200 mothers. Individuals were not intentionally recruited for both the qualitative and quantitative samples.

### Methods for quantitative survey

The survey inquired about the perinatal diet of mothers and ASHAs during their last pregnancy and in the first month post partum. Researchers collected data for 3 months from June to August 2019. The survey included yes/no questions about changes to the diet during and just after pregnancy, follow-up questions about each food item reported, the influencer associated with avoiding or adding it and the reason(s) for avoiding or adding it to the diet. Respondents could name multiple influencers and causes per food item. A specific sequence of questions proceeded as follows:

Did you avoid any food items during your pregnancy?

1. If yes, which food did you avoid? (free list response).
2. Who told you to avoid it? (multiple-choice options with an ‘other’ category available for additional write-in responses).
3. Why did you avoid it? (multiple-choice options were populated during the survey pilot phase and from the FGDs and KIIs, an ‘other’ category was available to capture additional write-in responses).

This structure was repeated for added foods during pregnancy, added foods during post partum and avoided foods post partum. The questionnaire also included demographic questions.

### Coding the qualitative discussions and quantitative survey

In total, FGD data processing resulted in 873 responses about added or avoided foods (410 from mothers and 463 from MIL). The KIIs resulted in 625 responses about added or avoided foods from influencers. These data were then tabulated to identify frequency by food item, type of recommendation (avoid or consume it) and timing concerning birth (during pregnancy or early post partum, although many recommendations pertained to both periods). All foods were coded according to the dietary diversity guidelines prescribed by the FAO: fruits, vegetables, dairy, meat, grains/tubers, eggs, pulses, nuts/seeds. To account for all items listed, we also added categories for coding including sweets (halwa), non-alcoholic beverages (tea) and an ‘other’ category (hot/spicy foods). Each food item was included in one food category. We also coded the purpose of the food-related practice: to promote health or avoid risk.

In total, quantitative survey responses were used to generate food lists for each of the four subsamples (add/avoid—pregnant/post partum). This generated 10 759 added or avoided food responses from mothers and 4959 added or avoided food responses from ASHAs, which were then consolidated to identify 315 unique food items from mothers and 254 from ASHAs. As with the food items in the qualitative data, each response was coded into food pyramid categories from the FAO.

## Results

We present the qualitative data first, followed by the quantitative data. Our mixed methods approach examines the role of a diverse set of health influencers operating within (and between) complex health systems in shaping beliefs about perinatal diet. Analyses for all methodologies included gathering frequency counts and running descriptive statistics by food item, type of recommendation (avoid or consume it), timing concerning birth (during pregnancy or early post partum), food group and purpose of food-related practice (to promote health or avoid risk). Many of the specific questions and option sets in the quantitative survey were designed and populated based on the results of the qualitative study. We also felt it necessary to distinguish novel qualitative data that are often overshadowed by quantitative data. This was done intentionally so that we could use the two studies to achieve convergence in depth and breadth.

For this reason, we consulted several possible sources of influence on the perinatal decisions and behaviours made by pregnant women in Bihar. A large sample for our qualitative data was not necessary because we did not make quantitative estimates of prevalence for each possible source of influence based on the qualitative data. Furthermore, we used KIIs rather than FGDs for some data collection because of the dispersion of people in different health influencer roles. For example, one village may have easier recruitment for multiple groups of four to five MILs while it is more difficult to assemble multiple groups of ANMs from both the researcher and participant perspectives. The KIIs were a pragmatic solution to certain health influencers being spread across larger geographic areas.

The quantitative survey provided a different kind of assessment for connections between influencer and behaviour than the qualitative discussions. The survey provides opportunities for estimated effect sizes and prevalence rates of certain associations. Many of the questions and option sets in the quantitative survey were directly based on results of the discussions from the qualitative discussions. We could not rely on existing research as the only source of information on which to base our quantitative survey because there are not sufficient data available on these topics. Furthermore, we did not want to risk omitting many locally important rituals and beliefs that are not captured empirically.

### Qualitative results

*Which added food items were most prevalent? What were the reasons for adding foods? Were there differences between influencers in dietary recommendations or justifications?*

For all participants, cow milk was the most commonly named food item to add to the perinatal diet, followed by pulses. Reasons for adding milk (often mixed with ghee) included easing labour during delivery and helping the body produce breast milk after birth, as well as statements about milk being nutritious or healthy. Pulses were associated with several general health-promoting benefits, while eggs were thought to warm the body, aid in breast milk production and to prevent bleeding. The most commonly stated reason across all of the participants for adding food to the diet is to promote health, including statements that the food promotes good health or strength of the mother and child, eases labour or helps the mother’s body to produce blood.

Sweet foods like pudding, Horlicks (a malted drink powder), rasgulla (sweet dumplings) and biscuits were frequently suggested dietary additions for delivery or post partum. Such sweet foods were associated with a range of potential benefits from easing labour and improving digestion and alleviating stomach problems. These recommendations align with the Ayurvedic recommendation to consume sweets during pregnancy to aid digestion.

*Which avoided food items were most prevalent? What were the reasons for avoiding foods? Were there differences between influencers in dietary recommendations or justification?*

For all participants, the most commonly named food to avoid was the wood apple, a popular acidic fruit native to India, followed by several other fruits: papaya, pineapple, banana, jackfruit and blackberry. Pickled foods and those that are salty, sour or spicy are also frequently avoided. While the wood apple is sometimes touted for its Ayurvedic benefits,^39^ the reason for avoiding it from multiple health influencers was primarily that it would cause ‘pus in the ear’ in the newborn. The majority of participants associated papaya and pineapple with miscarriage, which is consistent with Ayurvedic principles; papaya is considered a ‘hot’ food. Respondents across the spectrum of traditional to biomedical influencers reported that blackberries should be avoided during pregnancy because they turn a child’s lips black, a concern that was sometimes extended to other dark-coloured foods, like black carrots, or black foods in general. The following foods were avoided to prevent newborn pneumonia: banana, carrot, coconut water, cucumber, curds, guava, orange and rice. Foods such as coconut water, sweets and bananas were some of the foods mentioned to both add and avoid by different participants, leading to variation in recommendations. Many of the foods avoided post partum were omitted from the diet to prevent stomach pain and digestion issues in the baby. This includes not eating beans and avoiding salt because it could cause diarrhoea for the child.

### Quantitative results

*Which food items were added and avoided during pregnancy and post partum?*

Milk, apples and pomegranates were the most commonly named added foods, with leafy greens being higher ranked among ASHAs than among mothers (see tables 2 and 3). Horlicks, a sweet and processed dairy drink, was in the top 10 most named foods by mothers, but this is just 3.4% of the sample (Horlicks was not in the top 10 for ASHAs). During post partum, many of the added foods are sweet. The most frequently named food to avoid in both samples is brinjal (eggplant), and other seemingly healthy foods like banana, fish and mango are frequently avoided. A common reason for avoidance, particularly post partum, is that particular foods are oily and spicy.

**Table 2:** Top 10 frequently named foods by subsample, mother data

Rank	Pregnancy						Post partum					
	Add	n	%	Avoid	n	%	Add	n	%	Avoid	n	%
1	Pomegranate	511	17	Pickle	158	11	Pulses	534	16	Brinjal	422	14
2	Apple	507	17	Wood apple	124	8.9	Special halwa	534	16	Oily and spicy food	292	9.6
3	Milk	399	13	Yam	92	6.6	Milk	475	14	Curd	244	8
4	Coconuts/coconut water	205	6.9	Papaya	82	5.9	Jaggery	245	7.3	Pumpkin	229	7.5
5	Banana	175	5.9	Lemon	59	4.3	Ginger	229	6.9	Jackfruit	152	5
6	Orange	144	4.8	Fish	54	3.9	Black carom seeds	110	3.3	Banana	146	4.8
7	Grapes	131	4.4	Banana	53	3.8	Turmeric	106	3.2	Pickle	123	4
8	Horlicks	101	3.4	Jackfruit	46	3.3	Wheat/maize flour	98	2.9	Pigeon pea	98	3.2
9	Leafy vegetable	71	2.4	Blackberry	38	2.7	Rice	89	2.7	Fish	90	2.9
10	Beetroot	59	2	Mango	38	2.7	Warm water	85	2.5	Mango	88	2.9

**Table 3:** Top 10 frequently named foods by sample, ASHA data

Rank	Pregnancy						Post partum					
	Add	n	%	Avoid	n	%	Add	n	%	Avoid	n	%
1	Milk	204	13.9	Pickle	77	13.5	Milk	259	16.1	Brinjal	154	11.9
2	Apple	190	13	Wood apple	52	9.1	Special halwa	249	15.4	Pumpkin	152	11.7
3	Pomegranate	137	9.3	Yam	50	8.8	Pulses	209	13	Curd	130	10
4	Leafy vegetable	108	7.4	Blackberry	33	5.8	Ginger	99	6.1	Oily and spicy food	89	6.9
5	Banana	106	7.2	Papaya	26	4.6	Jaggery	90	5.6	Banana	80	6.2
6	Coconuts/coconut water	80	5.5	Banana	22	3.9	Black carom seeds	54	3.3	Pigeon pea	57	4.4
7	Orange	49	3.3	Jackfruit	22	3.9	Leafy vegetable	54	3.3	Pickle	51	3.9
8	Spinach	44	3	Lemon	22	3.9	Turmeric	53	3.3	Jackfruit	46	3.5
9	Pulses	43	2.9	Curd	17	3	Wheat/maize flour	50	3.1	Fish	34	2.6
10	Fish	39	2.7	Mango	16	2.8	Rice	40	2.5	Fava bean	30	2.3

ASHA, accredited social health activist.

*Which food categories were added and avoided during pregnancy and post partum?*

Fruit is the most commonly named food group for adding while pregnant, avoiding while pregnant and avoiding post partum, whereas sweets are the most commonly added food type post partum (see tables 4 and 5). Fruits are relatively rarely mentioned for adding post partum, ranked eighth and seventh for mothers and ASHAs, respectively, and less than 5% of the mentions in both cases.

**Table 4:** Food pyramid codes by subsample, mother data

Rank	Pregnancy						Post partum					
	Add	n	%	Avoid	n	%	Add	n	%	Avoid	n	%
1	Fruit	1796	60.2	Fruit	628	45.3	Sweets	893	26.8	Fruit	765	25
2	Dairy	446	15	Other	213	15.4	Pulses	541	16.2	Vegetable	674	22.1
3	Vegetable	275	9.2	Grains/tubers	136	9.8	Dairy	519	15.6	Other	597	19.5
4	Sweets	122	4.1	Vegetable	136	9.8	Vegetable	495	14.8	Pulses	273	8.9
5	Meat	90	3	Meat	120	8.7	Grains/tubers	306	9.2	Dairy	271	8.9
6	Nuts	79	2.6	Dairy	58	4.2	Beverage	144	4.3	Grains/tubers	255	8.3
7	Grains/tubers	68	2.3	Pulses	39	2.8	Nuts	123	3.7	Meat	171	5.6
8	Pulses	52	1.7	Eggs	36	2.6	Fruit	121	3.6	Sweets	36	1.2
9	Beverage	26	0.9	Sweets	9	0.6	Other	85	2.5	Eggs	7	0.2
10	Eggs	17	0.6	Beverage	8	0.6	Meat	75	2.2	Beverage	4	0.1
11	Other	11	0.4	Nuts	3	0.2	Eggs	32	1	Nuts	2	0.1

**Table 5:** Food pyramid codes by subsample, ASHA data

Rank	Pregnancy						Post partum					
	Add	n	%	Avoid	n	%	Add	n	%	Avoid	n	%
1	Fruit	673	45.9	Fruit	254	44.5	Sweets	387	24	Fruit	353	27.2
2	Vegetable	317	21.6	Other	101	17.7	Dairy	312	19.3	Vegetable	239	18.4
3	Dairy	233	15.9	Grains/tubers	73	12.8	Vegetable	274	17	Other	212	16.3
4	Meat	70	4.8	Vegetable	58	10.2	Pulses	212	13.1	Pulses	202	15.6
5	Pulses	53	3.6	Dairy	29	5.1	Grains/tubers	142	8.8	Dairy	139	10.7
6	Sweets	33	2.3	Meat	24	4.2	Nuts	68	4.2	Grains/tubers	89	6.9
7	Eggs	28	1.9	Pulses	13	2.3	Fruit	67	4.2	Meat	46	3.5
8	Grains/tubers	25	1.7	Eggs	8	1.4	Beverage	56	3.5	Sweets	9	0.7
9	Nuts	22	1.5	Beverage	6	1.1	Meat	40	2.5	Beverage	5	0.4
10	Other	9	0.6	Sweets	5	0.9	Other	37	2.3	Nuts	3	0.2
11	Beverage	3	0.2	Nuts	0	0	Eggs	18	1.1	Eggs	1	0.1

ASHA, Accredited Social Health Activist.

*Which health influencers are most commonly associated with perinatal food choice?*

**Figure 1:**
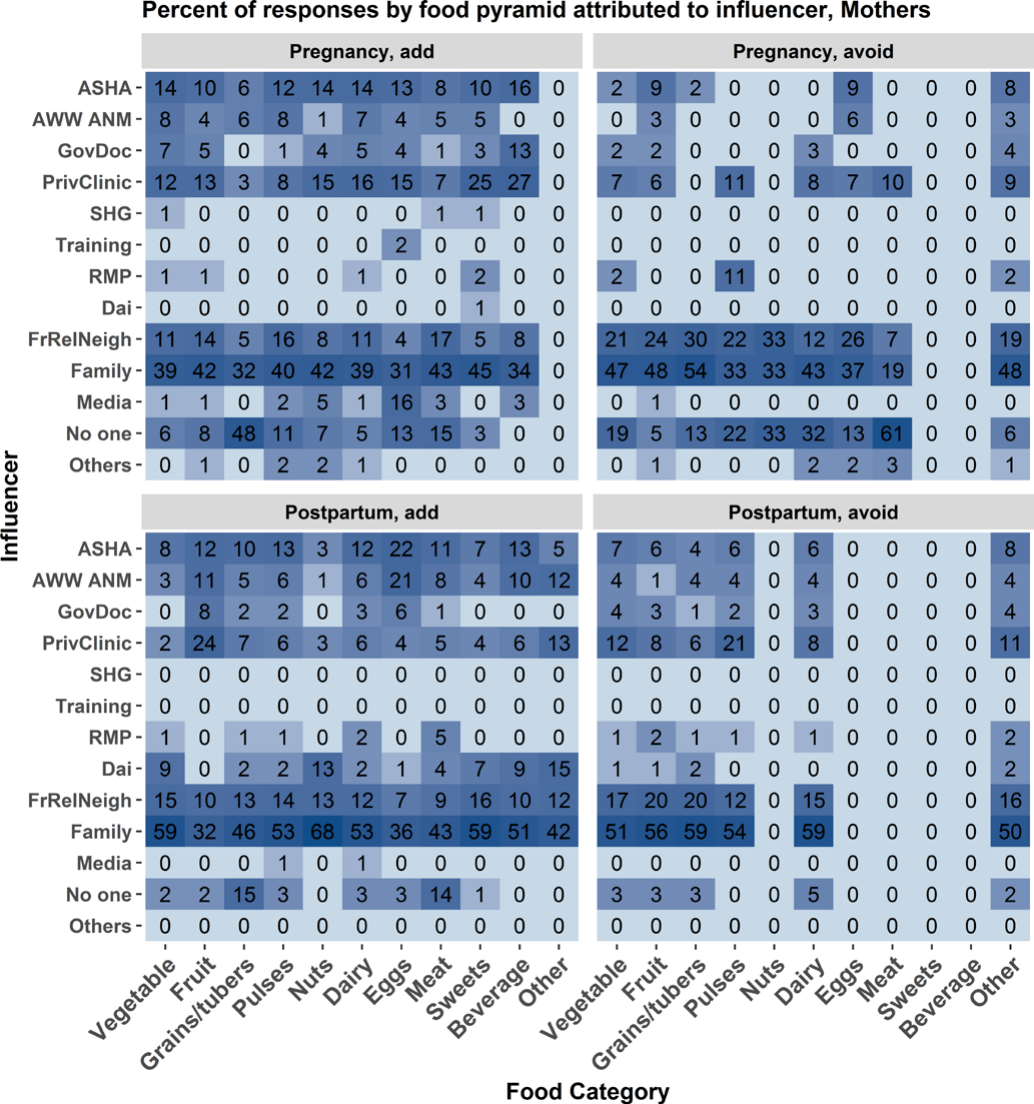
Mother data. The frequency of association between food category and source of influence for making food choices measured as a percent of total mentions associated with each source of influence. The associations are broken down by foods added during pregnancy (upper left), avoided during pregnancy (upper right), added post partum (lower left), and avoided post partum (lower right). Each cell represents the percent of times an influencer reason was associated with each food category. Darker blue shading indicates a higher percentage of mentions. Influencer category codes, y-axis: AWW ANM - Anganwadi Worker or Auxiliary Nurse Midwife; ASHA - Accredited Social Health Activist; FrRelNeigh - friends/relatives/neighbours; GovDoc - government doctor; PrivClinic - private clinic; RMP - Rural Medical Practitioner; SHG - self- help group.

**Figure 2:**
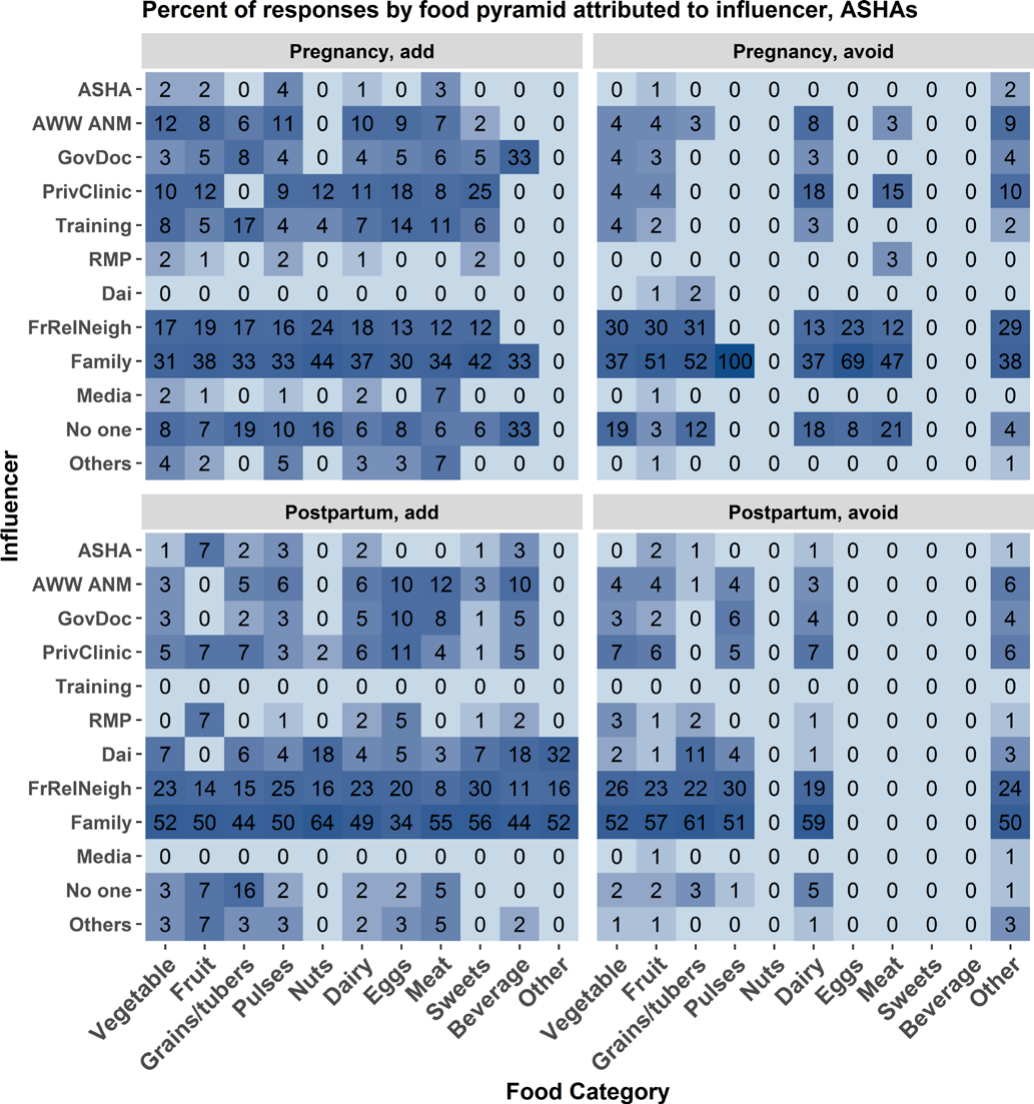
ASHA data. The frequency of association between food category and source of influence for making food choices measured as a percent of total mentions associated with each source of influence. The associations are broken down by foods added during pregnancy (upper left), avoided during pregnancy (upper right), added post partum (lower left), and avoided post partum (lower right). Each cell represents the percent of times an influencer reason was associated. Darker blue shading indicates a higher percentage of mentions. Influencer category codes, y-axis: AWW ANM - Anganwadi Worker or Auxiliary Nurse Midwife; ASHA - Accredited Social Health Activist; FrRelNeigh - friends/relatives/neighbours; GovDoc - government doctor; PrivClinic - private clinic; RMP - Rural Medical Practitioner; SHG - self- help group.

**Figure 3:**
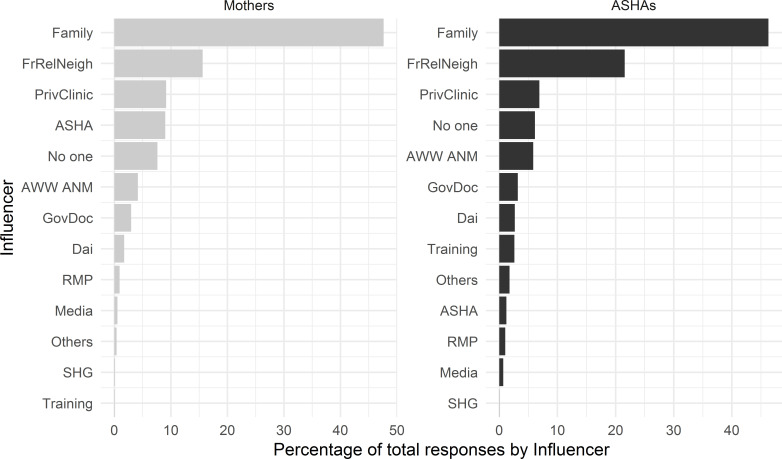
Bargraph showing overall frequency of mentions for each type of influencer in each sample, Mothers (left) and ASHAs (right). The frequencies represent total mentions across all foods to avoid or add during pregnancy and post partum. Influencer category codes, y-axis: AWW ANM - Anganwadi Worker or Auxiliary Nurse Midwife; ASHA - Accredited Social Health Activist; FrRelNeigh - friends/relatives/neighbours; GovDoc - government doctor; PrivClinic - private clinic; RMP - Rural Medical Practitioner; SHG - self- help group.

Figures 1 and 2 show, for each of 13 categories of individuals or groups influencing women’s dietary choices, how often that influencers recommended avoiding and how often they recommended adding each of 11 food groups—first considering pregnancy, and then considering the postpartum period. In all cases, the influencer categories of family and friends/relatives/neighbours were named more often than the CHWs. To determine which influencers were most commonly named for overall dietary consultation we summed the number of mentions across the subsamples and calculated one percentage for each influencer (figure 3). Family and the friends/relatives/neighbours categories are the most commonly named health influencers in both data sets, which would be consistent with previous studies. ASHAs receive training more often than mothers do in information-oriented services like nutrition education.

*Which reasons are most commonly associated with perinatal food choice?*

**Figure 4:**
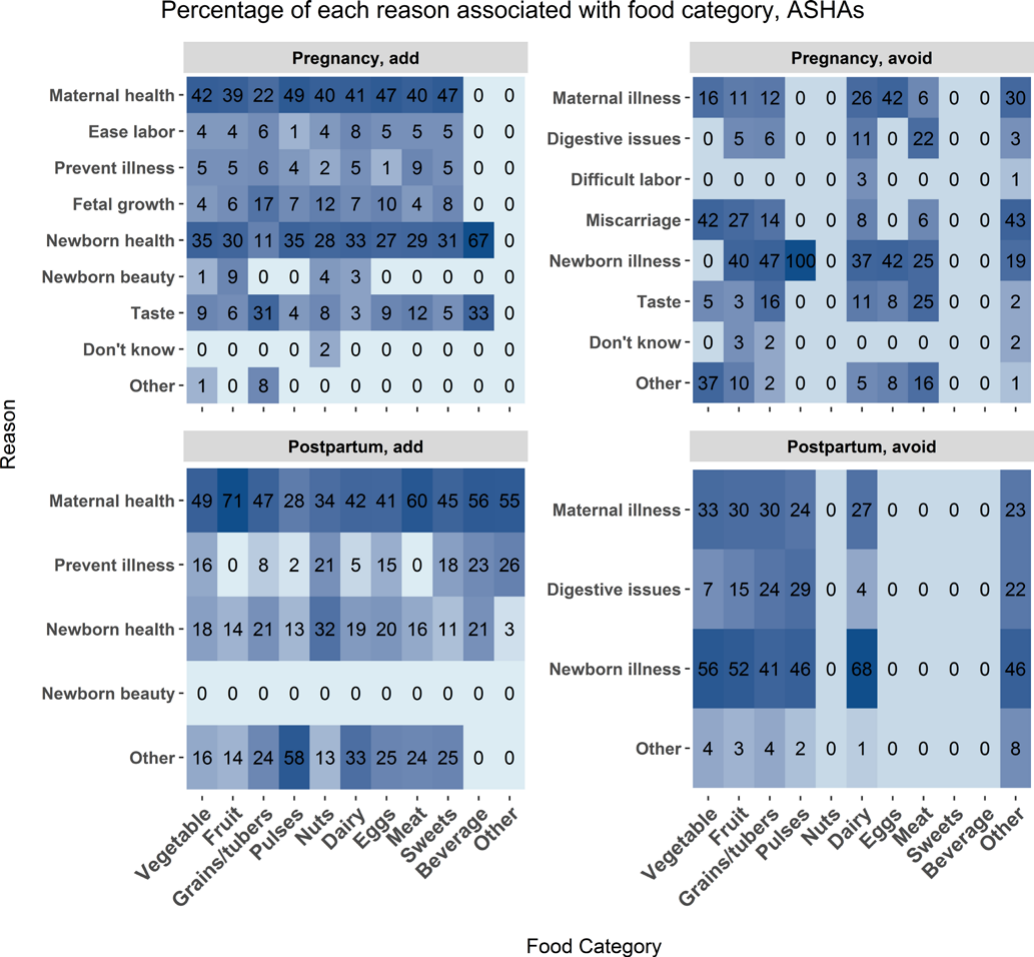
Mother data. The frequency of association between food category and food choice explanation measured as a percent of total mentions for each food category. The associations are broken down by foods added during pregnancy (upper left), avoided during pregnancy (upper right), added post partum (lower left), and avoided post partum (lower right). Each cell represents the percent of times a reason was associated with each food category. Darker blue shading indicates a higher percentage of mentions. Influencer category codes, y-axis: AWW ANM - Anganwadi Worker or Auxiliary Nurse Midwife; ASHA - Accredited Social Health Activist; FrRelNeigh - friends/relatives/neighbours; GovDoc - government doctor; PrivClinic - private clinic; RMP - Rural Medical Practitioner; SHG - self- help group.

**Figure 5:**
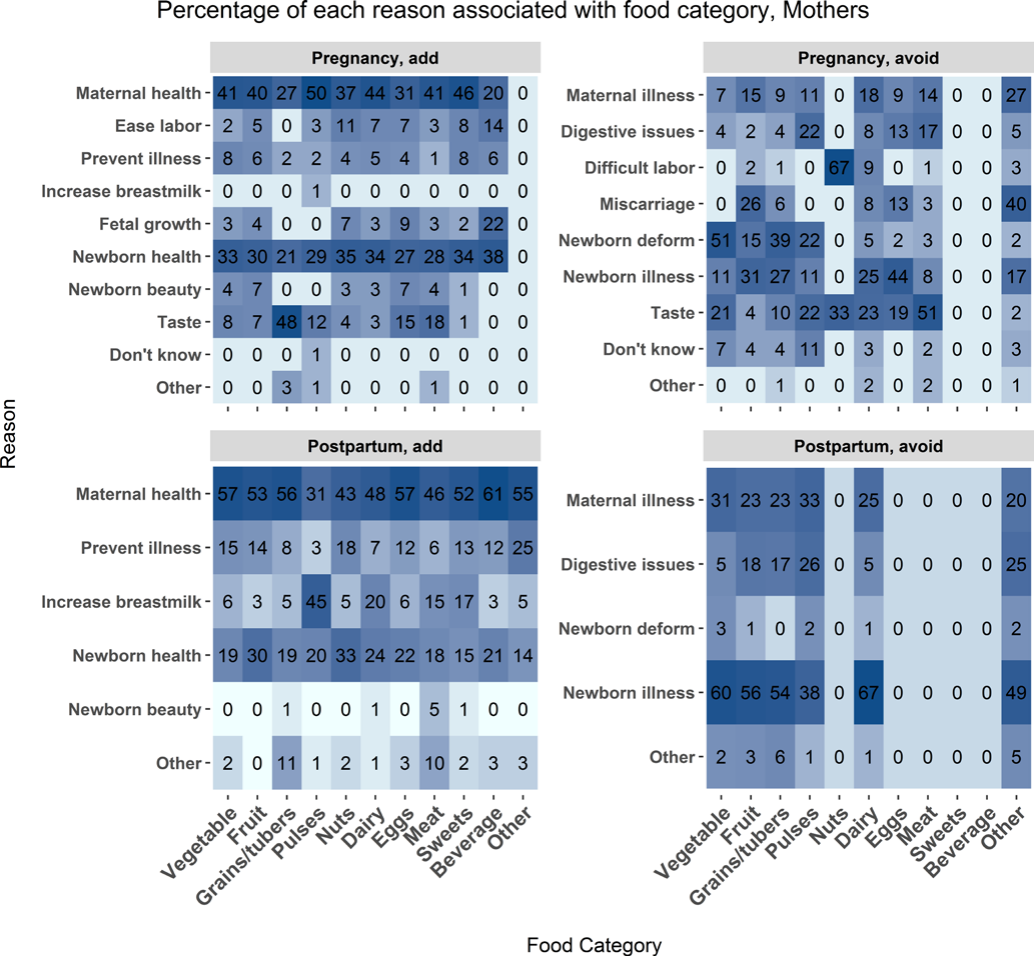
ASHA data. The frequency of association between food category and food choice explanation measured as a percent of total mentions for each food category. The associations are broken down by foods added during pregnancy (upper left), avoided during pregnancy (upper right), added post partum (lower left), and avoided post partum (lower right). Each cell represents the percent of times a reason was associated with each food category. Darker blue shading indicates a higher percentage of mentions. Influencer category codes, y-axis: AWW ANM - Anganwadi Worker or Auxiliary Nurse Midwife; ASHA - Accredited Social Health Activist; FrRelNeigh - friends/relatives/neighbours; GovDoc - government doctor; PrivClinic - private clinic; RMP - Rural Medical Practitioner; SHG - self- help group.

Overall, explanation distribution for food categories throughout the perinatal period by mothers and ASHAs is consistent as both added and avoided foods were justified based on promoting maternal and newborn health (see figure 4 and figure 5). Both ASHAs and mothers associated reasons for adding and avoiding foods such as coconuts, black carrots and blackberries with concerns of newborn appearance or deformity.

Taste was mentioned by mothers and ASHAs as a reason to add grain and avoid meat during pregnancy, which could also be a consequence of pregnancy-related symptoms like nausea. Mothers report concerns with preventing newborn deformities and promoting newborn beauty more frequently than ASHAs. As with the qualitative results, concern with digestive issues was common in both mothers and ASHAs.

*What is the association between explanation and influencer?*

**Figure 6:**
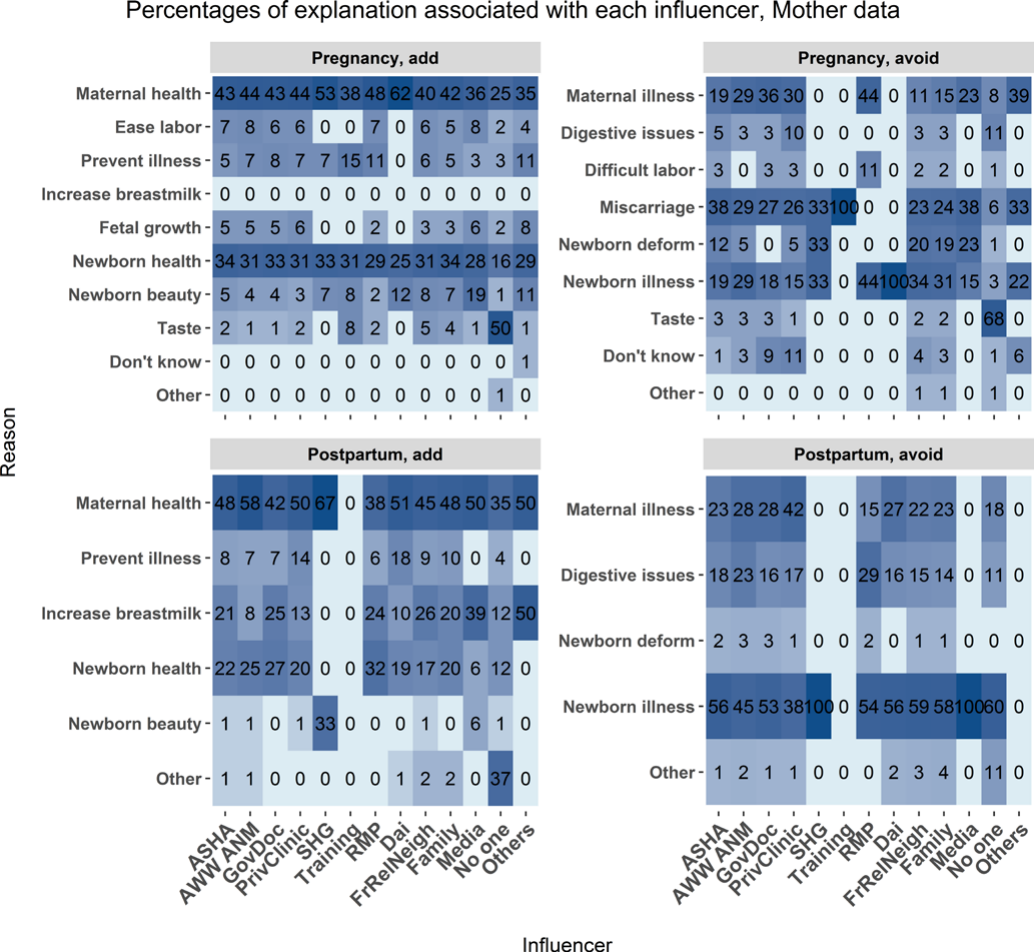
Mother data. The frequency of association between food choice explanation and source of influence measured as a percent of total mentions associated with each source of influence. The associations are broken down by foods added during pregnancy (upper left), avoided during pregnancy (upper right), added post partum (lower left), and avoided post partum (lower right). Each cell represents the percent of times a food choice explanation was associated with each influencer category. Darker blue shading indicates a higher percentage of mentions. Influencer category codes, y-axis: AWW ANM - Anganwadi Worker or Auxiliary Nurse Midwife; ASHA - Accredited Social Health Activist; FrRelNeigh - friends/relatives/neighbours; GovDoc - government doctor; PrivClinic - private clinic; RMP - Rural Medical Practitioner; SHG - self- help group.

**Figure 7:**
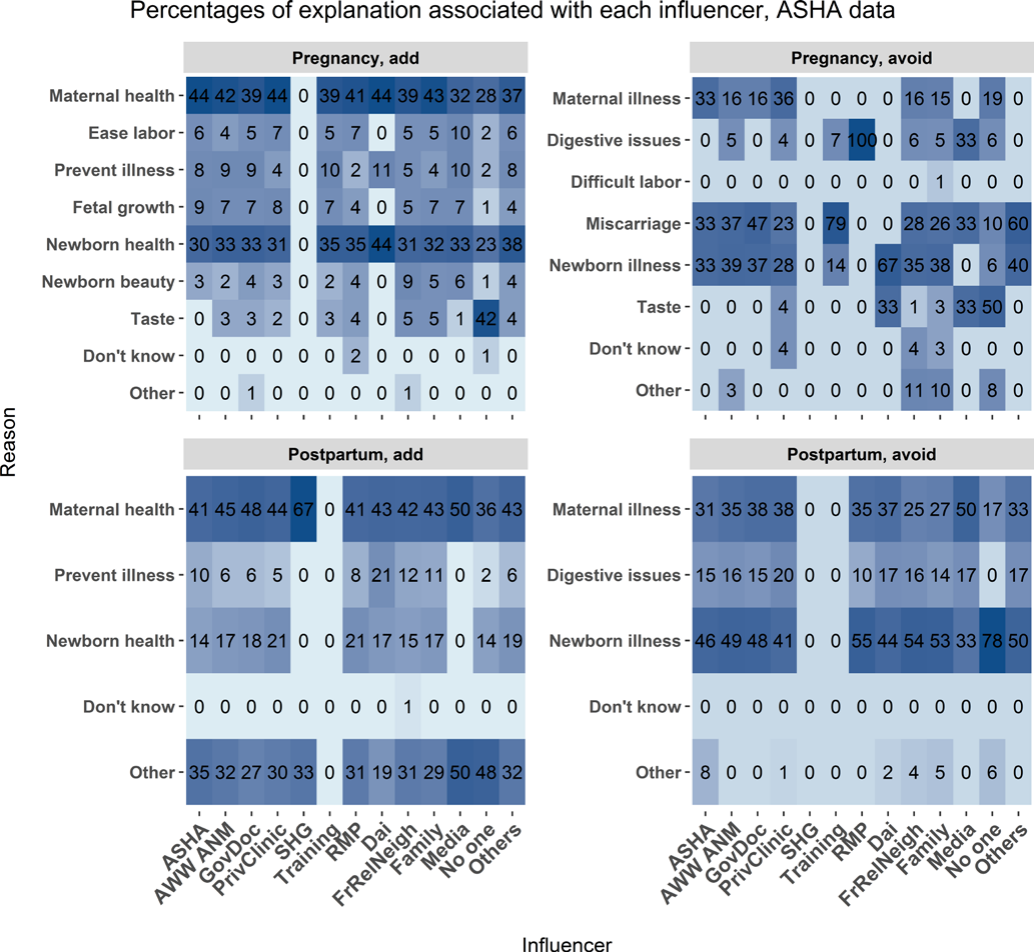
ASHA data. The frequency of association between food choice explanation and source of influence measured as a percent of total mentions associated with each source of influence. The associations are broken down by foods added during pregnancy (upper left), avoided during pregnancy (upper right), added post partum (lower left), and avoided post partum (lower right). Each cell represents the percent of times a food choice explanation was associated with each influencer category. Darker blue shading indicates a higher percentage of mentions. Influencer category codes, y-axis: AWW ANM - Anganwadi Worker or Auxiliary Nurse Midwife; ASHA - Accredited Social Health Activist; FrRelNeigh - friends/relatives/neighbours; GovDoc - government doctor; PrivClinic - private clinic; RMP - Rural Medical Practitioner; SHG - self- help group.

Mothers and ASHAs reported the influencers and the reasons associated with each food category, as summarised in the preceding sections. Figures 6 and 7 report the relations between the reason for the food choice and the influencer of the choice. Maternal health, newborn health, newborn illnesses and maternal illness were the most common explanations from all influencers. Both ASHAs and mothers added foods due to concerns with newborn beauty. Across most explanations, both mothers and ASHAs reported that the mother’s community (family, relatives, friends, neighbours) is a major influencer in their dietary choices.

## Discussion

Our objective was to document and describe the gastroecology of maternal perinatal diet in Bihar, a region at high risk for perinatal morbidity and mortality. We used mixed methods to document dietary choices, sources of dietary recommendations and explanations of nutrition-related beliefs during pregnancy and post partum among mothers, CHWs and other health influencers using FGDs, KIIs and quantitative surveys. In terms of the project’s design, the two sources closely complemented each other. The qualitative methods were invaluable for shaping the quantitative survey and gaining general insights into nutritional beliefs and behaviours surrounding the perinatal period in Bihar.

To understand a gastroecology, it is critical to review biomedical and traditional health systems as one system may support behaviours that the other does not. Furthermore, biomedicine does not use the local knowledge, skill, beliefs and practices of a culture to provide health improvements and recommendations. Biomedicine relies on empirical evidence, genetics and the environment and does not focus on cultural belief systems.

Ayurvedic principles and practices provide unique recommendations for individual pregnant mothers to achieve a balanced state. A wide range of ‘cooling’, nutritious foods such as milk, apples, pulses and pomegranates are added to the pregnancy diet by mothers and ASHAs. A wide range of ‘cooling’, nutritious foods such as wood apple, papaya and pickled foods are also frequently avoided during the postpartum period. Many of the avoided foods are sour, salty and oily, which risk creating imbalance due to their heating properties and compromise digestion, according to Ayurvedic principles. Biomedical recommendation conflicts with some of the recommended avoided foods such as wood apples and papaya as they are high in nutrients such as vitamin A and vitamin C. Though pregnant women and mothers are adding fruits, dairy and vegetables to their diets, frequently added protein recommendations would be ideal to see given prevailing low levels of meat and egg consumption in Bihar.^10^ These findings can provide some support for complimentary and conflicting beliefs and behaviours in a community with complex health systems.

Many explanations were provided for adding and avoiding foods during pregnancy that give a unique insight into Bihar’s gastroecology. The majority of the explanations provided by mothers and CHWs for adding foods were motivated by a desire to improve the health of the baby and/or mother; however, aesthetic concerns of the newborn’s appearance were also frequently mentioned. Additionally, high-calorie foods such as sweets that are added to the perinatal diet are low in macronutrients but may be consistent with the metabolic demands of pregnancy and lactation.^20^ The majority of explanations provided by mothers and CHWs for avoiding foods were motivated by a desire to avoid infections, avoid miscarriage or increase desired physical traits. Avoiding foods based on concerns over digestive issues was commonly mentioned, which reflects Ayurvedic principles concerned with proper digestion in the agni and may be functionally tied to concerns about shortages of clean water to sufficiently clean fruits and vegetables.^40^

### Health influencers

Consistent with previous research, families, relatives and neighbours are the primary influencers of dietary choice among mothers and ASHAs.^41^ This suggests that CHWs must deliver nutritional recommendations and interventions to multiple key family and community members and not just to mothers due to strong health influencer prevalence in perinatal dietary decision-making. Moreover, the quality of counselling to mothers can be improved by acknowledging other perinatal diet influencers. Additional support should also be directed towards educating CHWs about how to integrate local beliefs into maternal perinatal dietary recommendations.

### Coexistence and consistency of traditional and biomedical recommendations and practices

Many consistencies are found between traditional and biomedical health systems in Bihar and provide an opportunity to work towards synthesis in order to achieve better maternal health outcomes. For example, mothers and ASHAs reported adding foods such as fruits, dairy and grains/tubers to their diet throughout pregnancy and post partum for reasons related to Ayurveda practice which also align with biomedical recommendation. CHWs, including ASHAs, should present biomedical dietary recommendations in the context of existing gastroecologies, consisting of biomedical, traditional and family health influencers. CHW resources and training can integrate traditional and biomedical health beliefs in order to address locally relevant concerns, which range from fears about miscarriage to concerns about maternal recovery following birth.

We focus here on Ayurveda as an example of traditional medicine, yet there are many other traditional forms of medicine such as Unani and Siddha that are widespread in India. Furthermore, various cultural norms, practices and socioeconomic factors determine the nutritional status of women^42^ and may add discordance in dietary recommendations. For example, different mothers and ASHAs reported adding and avoiding the same foods, such as bananas, for reasons that are not directly linked to Ayurvedic practices.

## Concluding remarks

Multiple health systems influence maternal perinatal diet in Bihar. Nutritional interventions should be informed by knowledge of the complex coexistence of biomedicine and traditional medicine. Public health interventions should deliver educational content to mothers and relevant health influencers, including family members and CHWs. Knowledge of local gastroecologies will allow public health educators and CHWs to design educational interventions and messaging that (A) build on the strengths of local perinatal dietary behaviours and (B) address behaviours that could be detrimental to mother and child health outcomes.

## Data Availability

All data relevant to the study are included in the article.

## References

[1] World Health Organization . Who | World Health organization.

[2] McEwen BS , Wingfield JC . The concept of allostasis in biology and biomedicine. Horm Behav 2003;43:2–15. 10.1016/S0018-506X(02)00024-7 12614627

[3] Gelman SA , Legare CH . Concepts and folk theories. Annu Rev Anthropol 2011;40:379–98. 10.1146/annurev-anthro-081309-145822 23436950 PMC3579644

[4] Legare CH , Gelman SA , Bewitchment GS . Bewitchment, biology, or both: the co-existence of natural and supernatural explanatory frameworks across development. Cogn Sci 2008;32:607–42. 10.1080/03640210802066766 21635349

[5] Watson-Jones RE , Busch JTA , Harris PL , et al . Does the body survive death? Cultural variation in beliefs about life everlasting. Cogn Sci 2017;41 Suppl 3:455–76. 10.1111/cogs.12430 27859566 PMC10676006

[6] Watson-Jones RE , Busch JTA , Legare CH . Interdisciplinary and cross-cultural perspectives on explanatory coexistence. Top Cogn Sci 2015;7:611–23. 10.1111/tops.12162 26350158

[7] Legare CH , Akhauri S , Chaudhuri I , et al . Perinatal risk and the cultural ecology of health in Bihar, India. Philos Trans R Soc Lond B Biol Sci : 2020;375:20190433. 10.1098/rstb.2019.0433 32594881 PMC7423251

[8] Chakrabarti S , Chakrabarti A . Food taboos in pregnancy and early lactation among women living in a rural area of West Bengal. J Family Med Prim Care 2019;8:86–90. 10.4103/jfmpc.jfmpc_53_17 30911485 PMC6396620

[9] Placek CD , Madhivanan P , Hagen EH . Innate food aversions and culturally transmitted food taboos in pregnant women in rural Southwest India: separate systems to protect the fetus? Evol Hum Behav 2017;38:714–28. 10.1016/j.evolhumbehav.2017.08.001 29333059 PMC5764174

[10] Unisa S , Saraswat A , Bhanot A , et al . Predictors of the diets consumed by adolescent girls, pregnant women and mothers with children under age two years in rural eastern India. J Biosoc Sci 2020:1–20. 10.1017/S0021932020000462 32782055

[11] National family health survey (NFHS-4) India 2015-2016 Bihar 2017.

[12] Census of India Website : Office of the Registrar General & Census Commissioner, India. Available: https://censusindia.gov.in/vital_statistics/SRS_Bulletins/Bulletins.html [Accessed 5 Oct 2020].

[13] Nandan D . National rural health mission: turning into reality. Indian J Community Med 2010;35:453–4. 10.4103/0970-0218.74338 21278861 PMC3026119

[14] Sachdev Y , Dasgupta J . Integrated child development services (ICDs) scheme. Med J Armed Forces India 2001;57:139–43. 10.1016/S0377-1237(01)80135-0 27407319 PMC4925843

[15] Kosec K , Avula R , Holtemeyer B , et al . Predictors of essential health and nutrition service delivery in Bihar, India: results from household and frontline worker surveys. Glob Health Sci Pract 2015;3:255–73. 10.9745/GHSP-D-14-00144 26085022 PMC4476863

[16] Coffey D , Spears D . Neonatal Death in India : Birth Order in a Context of Maternal Undernutrition, 2019. 10.1093/ej/ueab028 PMC11244657 39005945

[17] International Institute for Population Sciences . National family health Survey-5, state fact sheet Bihar, 2019-2020: India.

[18] Hirvonen K , Bai Y , Headey D , et al . Affordability of the EAT-Lancet reference diet: a global analysis. Lancet Glob Health 2020;8:e59–66. 10.1016/S2214-109X(19)30447-4 31708415 PMC7024996

[19] Sharma M , Kishore A , Roy D , et al . A comparison of the Indian diet with the EAT-Lancet reference diet. BMC Public Health 2020;20:812. 10.1186/s12889-020-08951-8 32471408 PMC7260780

[20] Kominiarek MA , Rajan P . Nutrition recommendations in pregnancy and lactation. Med Clin North Am 2016;100:1199–215. 10.1016/j.mcna.2016.06.004 27745590 PMC5104202

[21] Centers for Disease Control and Prevention . Maternal diet | breastfeeding | CDC, 2020. Available: https://www.cdc.gov/breastfeeding/breastfeeding-special-circumstances/diet-and-micronutrients/maternal-diet.html

[22] Food and Agriculture Organization of the United Nations . Food Agric. organ. U. N. Available: http://www.fao.org/nutrition/education/food-dietary-guidelines/home/en/ [Accessed 6 Dec 2020].

[23] Darnton-Hill I , Mkparu UC . Micronutrients in pregnancy in low- and middle-income countries. Nutrients 2015;7:1744–68. 10.3390/nu7031744 25763532 PMC4377879

[24] Mousa A , Naqash A , Lim S . Macronutrient and micronutrient intake during pregnancy: an overview of recent evidence. Nutrients 2019;11. 10.3390/nu11020443. [Epub ahead of print: 20 Feb 2019]. PMC6413112 30791647

[25] Nazik E , Eryilmaz G . Incidence of pregnancy-related discomforts and management approaches to relieve them among pregnant women. J Clin Nurs 2014;23:1736–50. 10.1111/jocn.12323 24028734

[26] Iradukunda F . Food taboos during pregnancy. Health Care Women Int 2020;41:159–68. 10.1080/07399332.2019.1574799 30998436

[27] Ravishankar B , Shukla VJ . Indian systems of medicine: a brief profile. Afr J Tradit Complement Altern Med 2007;4:319–37. 10.4314/ajtcam.v4i3.31226 20161896 PMC2816487

[28] Pandey MM , Rastogi S , Rawat AKS . Indian traditional Ayurvedic system of medicine and nutritional supplementation. Evid Based Complement Alternat Med 2013;2013:1–12. 10.1155/2013/376327 PMC3705899 23864888

[29] Wells Y-O , Dietsch E . Childbearing traditions of Indian women at home and abroad: an integrative literature review. Women Birth 2014;27:e1–6. 10.1016/j.wombi.2014.08.006 25257377

[30] Matthews CM , Benjamin V . Health education evaluation and beliefs and practices in rural Tamil Nadu. 2. Family planning and antenatal care. Soc Action 1979;29:377–92. 12336272

[31] Nichter M , Nichter M . The Ethnophysiology and Folk Dietetics of Pregnancy: A Case Study from South India. In: Anthropology and international health. Springer Netherlands, 1989: 30–56.

[32] Pool R . Hot and cold as an explanatory model: the example of Bharuch district in Gujarat, India. Soc Sci Med 1987;25:389–99. 10.1016/0277-9536(87)90277-2 3317876

[33] Ramanamurthy PSV . Physiological effects of ‘hot’ and ‘cold’ foods in human subjects. J Nutr Diet 1969;6:187–91.

[34] Nag M . Beliefs and Practices about Food during Pregnancy: Implications for Maternal Nutrition Author(s): Moni Nag Source: Economic and Political, 1994.

[35] Puri S , Kapoor S . Taboos and myths associated with womens health among rural and urban adolescent girls in Punjab. Indian J Community Med 2006;31.

[36] Guha A . Ayurvedic concept of food and nutrition, 2006.

[37] Nanal VRM . Food in pregnancy an Ayurvedic overview. Anc Sci Life 2008;28:30–2. 22557294 PMC3336338

[38] Henrich J , Henrich N . The evolution of cultural adaptations: Fijian food taboos protect against dangerous marine toxins. Proceedings of the Royal Society B: Biological Sciences. Royal Society, 2010:3715–24. 10.1098/rspb.2010.1191 PMC2992713 20667878

[39] Jayakumar A , Geetha K . Wood apple: uses and benefits, 2012.

[40] India State-Level Disease Burden Initiative Collaborators . Nations within a nation: variations in epidemiological transition across the states of India, 1990-2016 in the global burden of disease study. Lancet 2017;390:2437–60. 10.1016/S0140-6736(17)32804-0 29150201 PMC5720596

[41] Coffey D . Prepregnancy body mass and weight gain during pregnancy in India and sub-Saharan Africa. Proc Natl Acad Sci U S A 2015;112:3302–7. 10.1073/pnas.1416964112 25733859 PMC4371959

[42] Rao KM , Balakrishna N , Arlappa N , et al . Diet and nutritional status of women in India. J Hum Ecol 2010;29:165–70. 10.1080/09709274.2010.11906259

